# Translation of SBGN maps: Process Description to Activity Flow

**DOI:** 10.1186/1752-0509-7-115

**Published:** 2013-10-31

**Authors:** Torsten Vogt, Tobias Czauderna, Falk Schreiber

**Affiliations:** 1 Leibniz Institute of Plant Genetics and Crop Plant Research (IPK) Gatersleben, Corrensstrasse 3, 06466 Stadt Seeland OT Gatersleben, Germany; 2 Institute of Computer Science, Martin Luther University Halle-Wittenberg, Von-Seckendorff-Platz 1, 06120 Halle (Saale), Germany; 3 Clayton School of Information Technology, Monash University, Victoria 3800, Australia

**Keywords:** SBGN, Translation, Process Description, Activity Flow

## Abstract

**Background:**

The Systems Biology Graphical Notation (SBGN) provides standard graphical languages for representing cellular processes, interactions, and biological networks. SBGN consists of three languages: Process Descriptions (PD), Entity Relationships (ER), and Activity Flows (AF). Maps in SBGN PD are often large, detailed, and complex, therefore there is a need for a simplified illustration.

**Results:**

To solve this problem we define translations of SBGN PD maps into the more abstract SBGN AF maps. We present a template-based translation which allows the user to focus on different aspects of the underlying biological system. We also discuss aspects of laying out the AF map and of interactive navigation between both the PD and the AF map. The methods developed here have been implemented as part of SBGN-ED ( http://www.sbgn-ed.org).

**Conclusions:**

SBGN PD maps become much smaller and more manageable when translated into SBGN AF. The flexible translation of PD into AF and related interaction methods are an initial step in translating the different SBGN languages and open the path to future research for translation methods between other SBGN languages.

## Introduction

### SBGN

To standardise graphical representations of biological processes and cellular interactions, a broad community of biologists, curators, modellers, and software developers designed the Systems Biology Graphical Notation (SBGN) [1]. The main aim of SBGN is to provide a uniform set of symbols (called glyphs) to present biological networks and processes in an unambiguous manner and therefore to ease the exchange of biological knowledge. SBGN defines three different languages: the Process Description language (PD), the Entity Relationship language (ER), and the Activity Flow language (AF). Each of these languages shows the information of the represented biological process as a SBGN map in a different way, focusing on different aspects and different levels of granularity and therefore providing a complementary view on the underlying biological system. All of them have a set of glyphs to represent different entities or activities, and arcs to represent the interactions between these glyphs.

*PD* maps show how entities are processed into each other in the network and their influence upon reactions, *ER* maps focus on the influence which entities have upon the behaviour of others, and *AF* maps are used to show the flow of activities from one entity to another in a more abstract and often ambiguous way.

**Figure 1:**
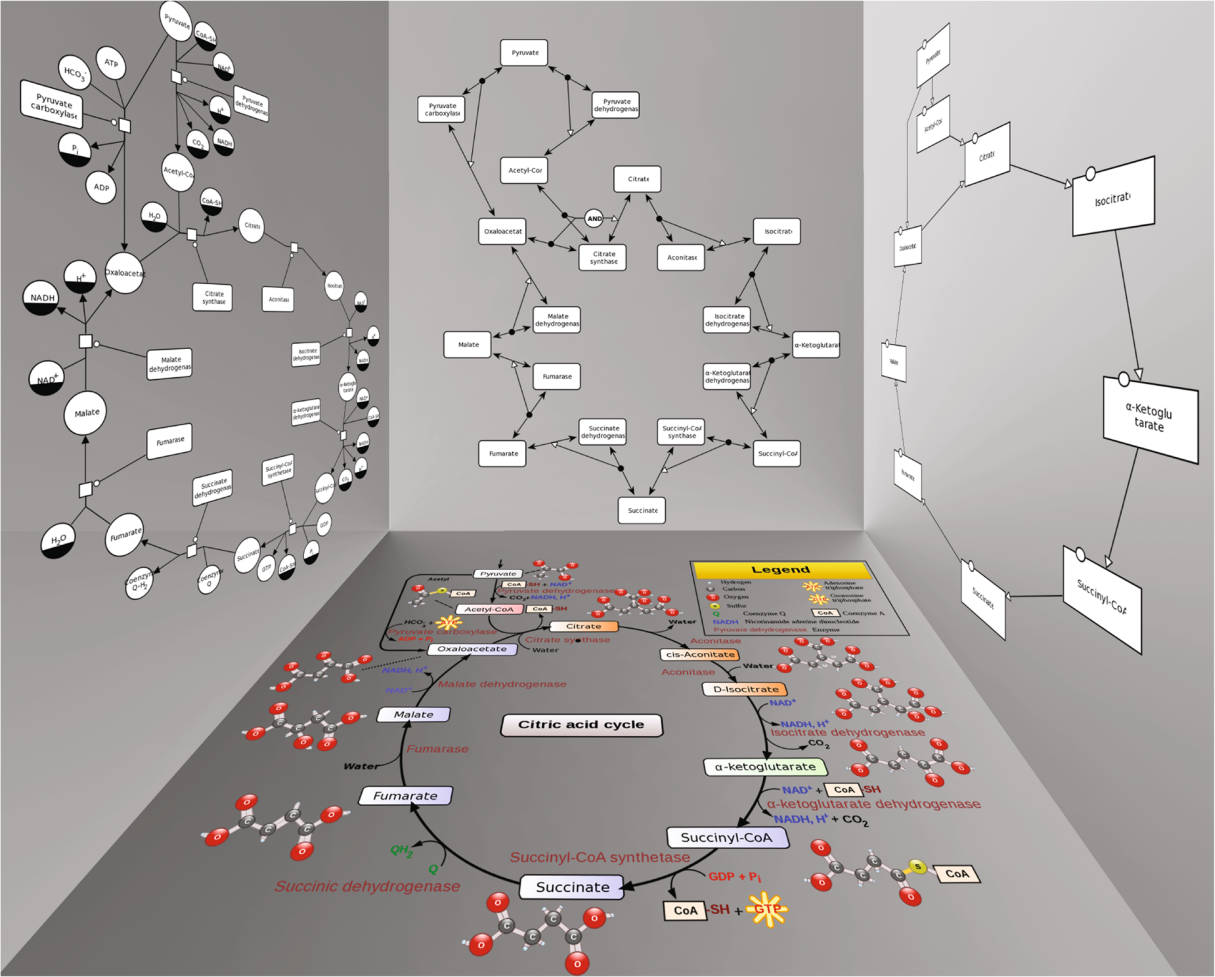
**The citric acid cycle.** An example of how the three different SBGN languages could represent a biological pathway. Bottom: schematic visualization of the citric acid cycle [2,3], left: PD view of the pathway, top: ER view of the pathway, right: AF view of the pathway.

In general, the three languages correspond to three levels of abstraction of the same biological knowledge which makes the translation between the languages possible and meaningful. For an example of the three languages see Figure 1[2,3]. However, in particular the PD language is suitable for the representation of knowledge from biochemistry (e. g., metabolic networks), the ER language can be used to present knowledge from molecular biology (e. g., protein interaction networks), and the AF language is suitable to show physiological knowledge.

### SBGN-ED and VANTED

VANTED [4,5] is an open source software for the analysis, modelling and visualisation of different omics data in the context of biological networks. VANTED allows the loading and editing of graphs, the mapping of experimental data sets onto the graph elements, the visualisation of time series data or data of different genotypes in the context of the underlying biological processes, and the analysis of the data. Extensions based on VANTED address, for example, metabolic modelling, an information system for experimental data, and investigation of images and volumes. SBGN-ED [6,7] is a VANTED add-on which allows the creation and editing of all three types of SBGN maps, the validation of these maps according to the SBGN specifications, the translation of maps from the KEGG pathway database into SBGN, and the export of SBGN maps into several file and image formats.^a^

### Motivation

Maps in SBGN Process Description (PD) are often too big and too complex, so there is a need for a simplified illustration. One way to solve this problem is a translation into the more abstract SBGN Activity Flow (AF) language which provides a more abstract view of the biological processes. However, the abstraction intrinsically leads to a loss of information during the translation of PD maps into AF maps.

Since different users may want to emphasise different information in their maps, a fully automatic translation may not be flexible enough, and we will argue that a template-based semi-automatic translation may be a better solution. The templates provide specific translation rules which are either predefined or given by the user. We will discuss the translation process and present an implementation of the method in SBGN-ED. Four templates are optimised for different translation types. For example, a user could focus on enzyme activities or on metabolite flow.

The problem discussed in this paper is different to two other important tasks: 1) the translation of information about biological networks, cellular processes and mathematical models into the graphical SBGN representation, and 2) the translation of mathematical models between different representations. For 1) there is ongoing work concerning the translation of widely used languages such as SBML, BioPax, and KGML (the XML-representation of the graphical KEGG maps) into SBGN maps, and initial translation methods are available such as KEGG to SBGN [8]. Examples for 2) are the translation of relations from BioPAX to SBML [9] and the translation of reaction graphs to activation/inhibition graphs [10]. However, a SBGN map is just some graphical representation of knowledge and should not be misinterpreted as an executable mathematical model of a biological process. This paper is neither concerned with the translation of mathematical models into SBGN maps nor with different modelling approaches for simulation or analysis purposes but focuses on the translation of the graphical information from one SBGN language to another.

In the following this paper provides more background to the PD and AF languages, discusses the translation process, presents an implementation in SBGN-ED, and finally shows examples of the template-based translation of SBGN PD maps to AF maps.

## Background

### The Process Description language

**Figure 2:**
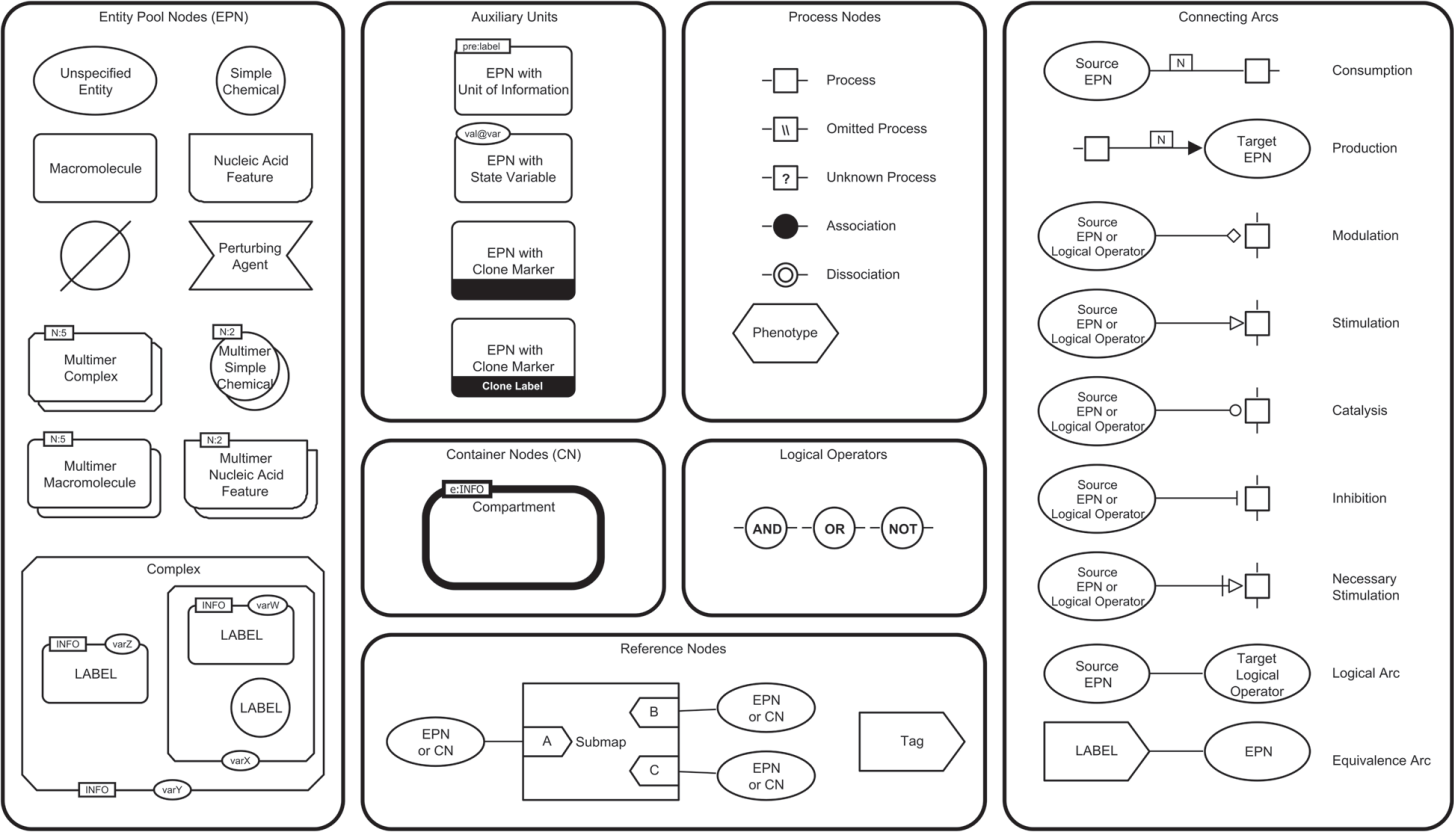
**Process Description reference card.** Process Description (PD) reference card reproduced from [11].

Figure 2 shows the Process Description (PD) language reference card. This section gives an overview of the different graphical elements of PD. Detailed descriptions, the grammar, and layout rules of PD can be found in the specification of the language [11].

#### Entity Pool Nodes (EPN)

An *entity pool* is a population of entities that cannot be distinguished from each other. For example, all molecule entities that fulfill the same role in a given process form an *entity pool*. An *entity pool* can represent different granularity levels, such as all the proteins or only certain forms of a protein. There are several types of EPNs: *unspecified entities*, *simple chemicals*, *macromolecules*, *nucleic acid features*, and *complexes*. In addition, there are glyphs for *multimer* representation (which is an aggregation of multiple identical or pseudo-identical entities held together by non-covalent bonds). PD also has three conceptual entities: *source*, *sink*, and *perturbing agent*. Biochemical networks can be affected by external influences represented by the *perturbing agent* glyph. *Source* and *sink* represent the possibilities to acquire or remove entities.

#### Auxiliary units

*Auxiliary units* are glyphs that enhance or decorate other glyphs and provide additional information. Examples are annotations (*unit of information*), state information (*state variable*), or duplications of EPNs (*clonemarker*). *Units of information* and *state variables* may be added multiple times to a glyph.

#### Process Nodes (PN)

*Process nodes* represent processes that transform one or more *entity pools* into one or more *entity pools*. Types are 

• *Process*: a generic process which transforms a set of *entity pools* into another

• *Omitted process*: a process which exists but is not shown on the map in detail

• *Uncertain process*: a process which may not exist

• *Association*: a non-covalent binding of objects

• *Dissociation*: a rupture of a non-covalent binding of objects

• *Phenotype*: a process which generates phenotypes.

#### Logical operators

SBGN PD contains the three logical operators *and*, *or*, and *not*.

#### Other nodes

SBGN PD contains some nodes which cannot be assigned to the previous categories. 

• *Compartment*: a logical or physical structure that contains *EPNs*

• *Submap* and *tag*: a structure to encapsulate processes and handle references to substructures

#### Arcs

Arcs are edges that connect *EPNs* and *PNs*. The symbols attached to their extremities indicate their semantics. 

• *Consumption*: the *entity pool* is consumed by the process

• *Production*: the *entity pool* is produced by the process

• *Stimulation*: the flux of the process is positively affected

• *Catalysis*: a particular case of stimulation

• *Inhibition*: the flux of the process is negatively affected

• *Necessary stimulation*: stimulation that is necessary for a process to take place

• *Modulation*: the flux of the process is affected positively, negatively or even in both ways

• *Logical arc*: represents the influence of an *EPN* to the outcome of a logic operator

• *Equivalence arc*: equivalence between *EPN* and tag

### The Activity Flow language

**Figure 3:**
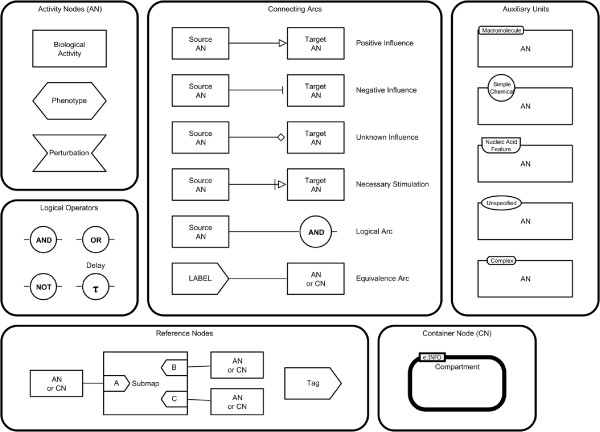
**Activity Flow reference card.** Activity Flow (AF) reference card reproduced from [12].

Figure 3 shows the Activity Flow (AF) language reference card. This section gives an overview of the different graphical elements of AF. Detailed descriptions, the grammar, and layout rules of AF can be found in the specification of the language [12].

#### Activity Nodes (AN)

An *activity node* represents the activity of an entity or an entity pool but not the entities themselves. SBGN AF uses one glyph to represent activities from all biological entities. The nature of the molecule that the activity comes from, such as *simple chemical* or *macromolecule*, can be encoded in the *units of information*. A biochemical network can generate phenotypes or affect processes which are represented by the *phenotype* glyph. It can also be affected by external influences which are represented by the *perturbation* glyph.

#### Auxiliary units

For biological activities the nature of the entity where the activity originates can be represented using *units of information*. Different symbols which are identical to the *entity pool node* symbols in SBGN PD are used.

#### Logical operators

SBGN AF contains the four *logical operators* *and*, *or*, *not*, and *delay* (which denotes that the *activity node* linked as input does not produce the influence immediately).

#### Other nodes

SBGN AF contains some nodes which can not be assigned to the previous categories.

• *Compartment*: a logical or physical structure that contains *EPNs*

• *Submap* and *tag*: a structure to encapsulate processes and handle references to substructures

#### Arcs

Arcs are edges that link *ANs* together. The symbols attached to their extremities indicate their semantics. 

• *Positive influence*: an action that produces a positive/activating effect from one activity to another

• *Negative influence*: an action that produces a negative/inhibiting effect from one activity to another

• *Unknown influence*: used when the effect exerted from one activity to another is not well understood

• *Necessary stimulation*: influence that has to be present for the target activity to take place

• *Logical arc*: used to indicate that an entity influences the outcome of a logic operator

• *Equivalence arc*: used to indicate that all entities marked by a tag are equivalent

## Methods

### Translation from PD to AF

There are many ways to translate a PD map to an AF map, depending on the level of granularity or different aspects of the map. Therefore a semi-automatic concept for the translation process was designed.

**Figure 4:**
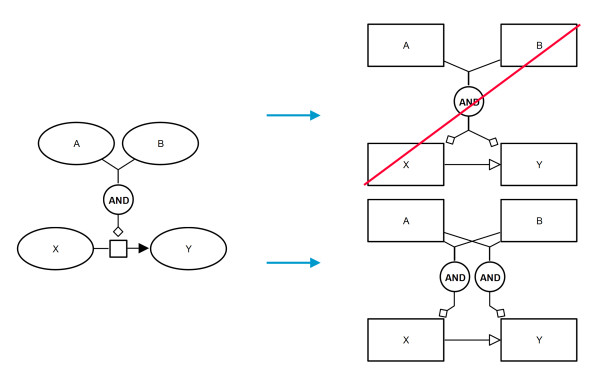
**Translation diagram for logical operators using the example of and.** Left: a possible case which could occur in a PD map; right: on top the invalid translation is shown (two outgoing arcs from the *and* operator are not allowed in SBGN) and at the bottom the correct translation.

The two languages have several common glyphs. This includes *compartment*, *phenotype*, *perturbating agent*, *submap*, *tag*, *and*, *or*, and *not* as well as *logical arcs* and *equivalence arcs*. These glyphs can be directly translated because they exist in both languages and have the same meaning. Hereby the logical operators represent a special case. In PD the target of a *logical operator* is always a *process node* (or another *logical operator*). As a result from a straightforward translation there could be multiple outgoing *modulating arcs* from the *logical operators*. This is not allowed in SBGN and hence the translation must generate multiple *logical operators* with the same ingoing arcs and exclusive outgoing arcs (see Figure 4).

#### Translation of nodes

**Figure 5:**
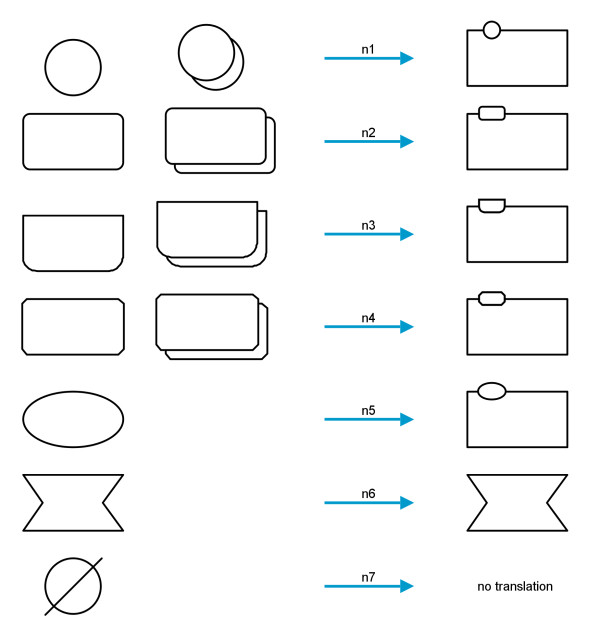
**Translation diagram for the entity pool nodes.** Left, from top to bottom: *simple chemical*, *macromolecule*, *nucleic acid feature*, *complex*, *unspecified entity*, *perturbating agent*, *source*/*sink*; center, from top to bottom: *multimer simple chemical*, *multimer macromolecule*, *multimer nucleic acid feature*, *multimer complex*; right: translated *activity nodes* with decorations.

SBGN PD also contains glyphs which have no corresponding glyph in SBGN AF and therefore have to be translated in a different way. The translation of *entity pool nodes* is straightforward. As a pendant for these in AF there are only the *activity nodes*, but *EPNs* have *auxiliary units* which are equal in shape and meaning to the *entity pool nodes*. Figure 5 shows how each *entity pool node* is translated. At this point some information may be lost because multimers cannot be represented in AF (translations n1 to n4). Furthermore the *auxiliary units* of the PD nodes are lost.

**Figure 6:**
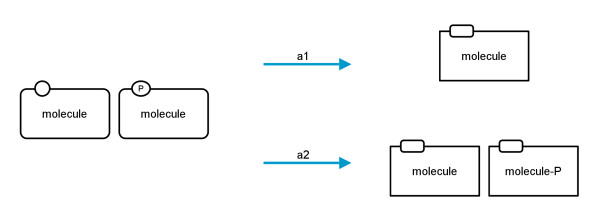
**Translation diagram showing the possibilities for translating nodes with the same label but different auxiliary units.** Left: two *macromolecules* with the same label but different *auxiliary units*; right top: fused node with discarded *auxiliary units*; right bottom: two nodes with concatenated *auxiliary unit* labels.

The PD language allows nodes with the same label and different *auxiliary units* in one *compartment*. In AF the *activity nodes* have only one *auxiliary unit* which defines the nature of the entity from which the activity comes. There are two options for translating nodes with the same label and different *auxiliary units*. Either they can be fused into one *activity node* and the old *auxiliary units* are discarded (translation a1), or they are translated independently and the labels of the *auxiliary units* are concatenated to the label of the nodes (translation a2). The two possibilities are depicted in Figure 6.

*Sink* and *source* nodes can not be translated as there are no corresponding glyphs in AF. However, this translation is also unnecessary as AF depicts the flow of activities and not the flow of the molecules themselves.

**Figure 7:**
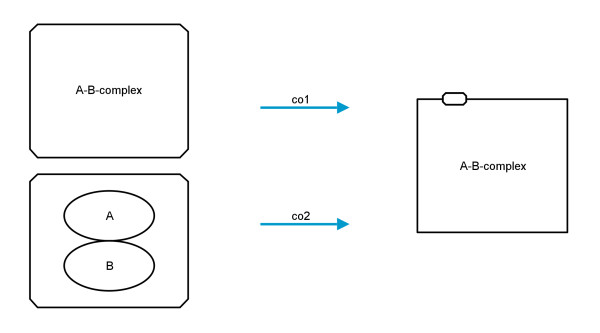
**Translation diagram for complexes.** Left: the two possible forms of *complexes* in PD, on top a complex with a label and at the bottom a complex with decorations; right: the only possible translation in AF.

Another *entity pool node* glyph is the *complex*. In PD *complexes* can be defined with a label and/or with other *entity pool nodes* as decorations (or description) of the *complex*. AF does not allow such complex decorations for *activity nodes*. If the PD node has a label, this is used as a label for the *activity node*, otherwise the label of the new AF node is created by concatenating the labels of the decorations of the PD *complex* (translations co1 and co2, see Figure 7).

**Figure 8:**
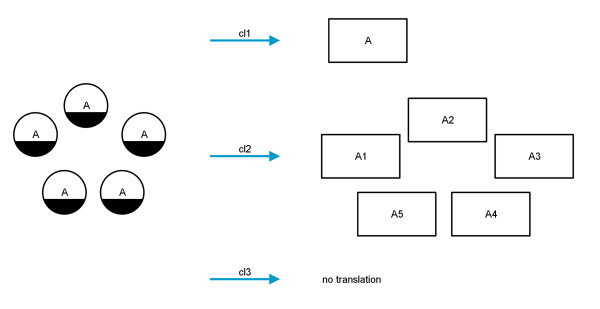
**Translation diagram for nodes with clonemarkers.** Left: *simple chemicals* in PD decorated with *clonemarkers*; right: three different possible translations; top: fusing all nodes into one node; middle: number the nodes sequentially; bottom: no translation.

Furthermore, nodes with *clonemarkers* increase the complexity of the translation process. In PD *clonemarkers* are used to represent *entity pool nodes* which occur several times in a *compartment*. The concept of clonemarkers is not available in AF. There are several possibilities for translating nodes with clonemarkers to AF (see Figure 8). First, it is possible to fuse the nodes and to bundle all of their arcs into this new node (translation cl1), but the graphical representation could become confusing or unclear depending on the size and the density of the map. Second, the nodes with *clonemarkers* may be deleted (translation cl3). In many cases co-factors, such as ATP, appear several times on a map and therefore have *clonemarkers*. These co-factors are not always necessary for the flow of information and may be deleted for reasons of clarity. The third possibility for the translation is to number the nodes serially and to add the number to the label of the node (translation cl2). This method should only be used when the map is edited afterwards and the labels are changed manually since the grammar of the AF language would be disregarded. The translation of nodes with *clonemarkers* is shown in Figure 8.

#### Translation of arcs

*Logical arcs* and *equivalence arcs* can be translated directly. The translation of other arcs is not straightforward. These arcs are connected to the different types of *process nodes* (in the following the glyph *phenotype* is ignored for now). These *process nodes* are central glyphs of PD maps as they represent all reactions which take place in the biological system. *Process nodes* always have at least one incoming *consumption* arc and one outgoing *production* arc or, in case of a reversible reaction, at least one *production* arc incoming and outgoing. This implies that a relation can be set between both sides of the reaction. In addition one or more modulating arcs can be connected to each *process node*. The modulating arcs affect the reaction and therefore they are also in relation to both sides of the reaction but they do not affect each other.

**Figure 9:**
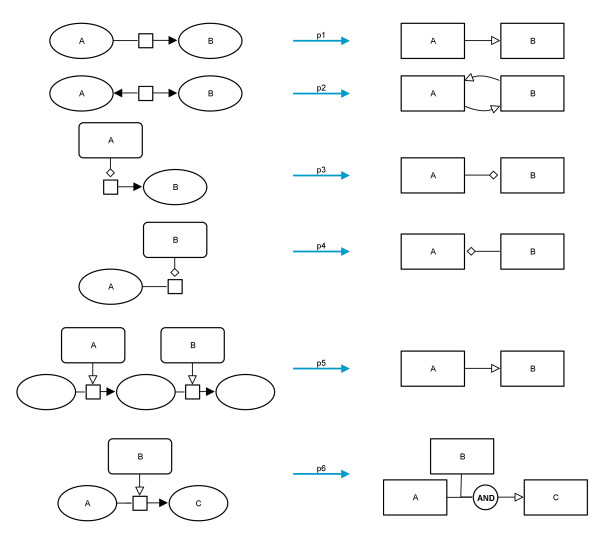
**Possible relations between arcs and a potential translation for each.** From top to bottom: relation between consumption and production, relation between productions, relation between modulation and production, relation between modulation and consumption, relation between two neighbouring modulations (here: stimulations) of the map which share a common substrate/product, consideration of all elements of a reaction.

How these relations appear in the SBGN PD language and how they might be translated is shown in Figure 9 (translations p1 to p4). In addition Figure 9 shows two further translation possibilities. Translation p5 can be used to show the relations between neighbouring modulations which share a common substrate/product, and translation p6 can be used to represent the fact that all substrates and modifiers are needed for this process.

**Figure 10:**
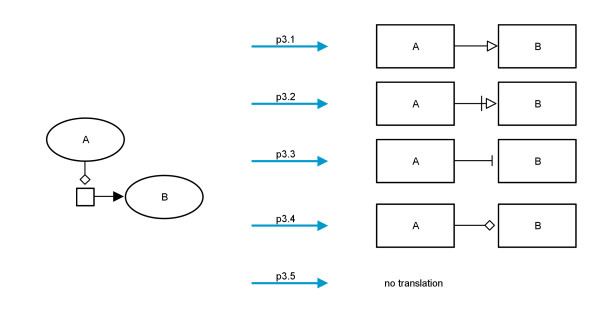
**Translation diagram showing the possibilities for translating process nodes with the relation modulation ↔ production.** For each relation within the network (modulating arc ↔ *consumption* or modulating arc ↔ *production*) the user can choose if and how they should be translated.

Each kind of modulating arc has a specific translation. In many cases the translation is straightforward, i. e., a *stimulation* should be translated into a *positive influence*, but there are other cases in which it is not quite clear. A *catalysis* can be translated into a *positive influence*, but sometimes it could also be seen as a *necessary stimulation*, because under normal conditions most reactions do not process without the appropriate enzyme. The relation *modulation* ↔ *consumption* might be translated into a *negative influence*, but also the translation to an *unknown influence* is worth considering. Different possibilities for the relation *modulation* ↔ *production* are shown in Figure 10.

Table 1 contains some recommendations on the translation of the different modulating arcs.

**Table 1:** Recommendations for the translation of arcs

**PD** ***modulating arc***	***Modulating arc* →** ***consumption***	***Modulating arc* →** ***production***
*Modulation*	*Unknown influence*	*Unknown influence*
*Stimulation*	*Unknown influence*, *negative influence*	*Positive influence*
*Catalysis*	*Unknown influence*, *negative influence*	*Positive influence*
*Inhibition*	*Unknown influence*, *positive influence*	*Negative influence*
*Necessary stimulation*	*Unknown influence*, *negative influence*	*Necessary stimulation*

### Translation algorithm

In the first step the *entity pool nodes* of the PD map are translated to the corresponding *activity nodes* for the AF map. Nodes with the same label and different *auxiliary units* will be translated according to the choices made by the user. Depending on the chosen method, nodes with a *clonemarker* will be either combined into one *biological activity*, or numbered serially, or deleted completely.

The second step of the translation algorithm is the translation of the arcs. Here the *process nodes* are the central nodes of PD maps, hence they are traversed and tested as to whether a translation rule has to be applied.

In the last step it may be necessary to revise the created AF map for invalid or redundant glyphs. For example, *logical operators* might have multiple outgoing modulating arcs because their target *process node* has multiple incoming and outgoing arcs. In these cases the *logical operator* with all its incoming arcs is multiplied so that each has exactly one outgoing modulating arc. Furthermore, the *logical operators and* and *or* might have only one incoming arc. This can happen because nodes were deleted from the map during the translation. These logical operators are invalid and are deleted from the map during the revision, and the source glyph and target glyph of their arcs are connected directly.

### Translation templates

**Figure 11:**
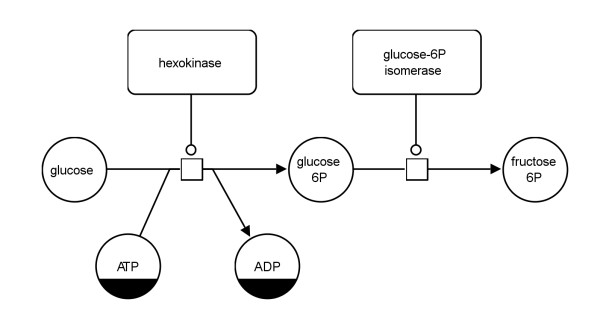
**Example of a Process Description map showing the first two reactions of glycolysis (see also Figure **13** top).**

In the previous sections several rules for the translation of SBGN PD maps to SBGN AF maps have been described. These rules provide the possibility for a dynamic translation which can be adapted to the required results. However, four translation templates have been defined which combine translation rules for common use cases of SBGN PD to AF translations. These templates will be presented in the following subsections using the PD map in Figure 11 as an example.

#### Enzyme activities template

The *enzyme activities* template shows the influences which the enzymes have on the metabolites.

In case of irreversible reactions this template produces a modulating arc in AF corresponding to the modulating arc in PD in the production direction of the process (e.g., a *positive influence* for a *catalysis* or a *stimulation*, a *negative influence* for an *inhibition*, an *unknown influence* for a *modulation*, and a *necessary stimulation* for a *necessary stimulation*) and an *unknown influence* in the consumption direction.

In case of reversible reactions the same rule is applied in both directions of the process as for the production direction of irreversible reactions.

**Figure 12:**
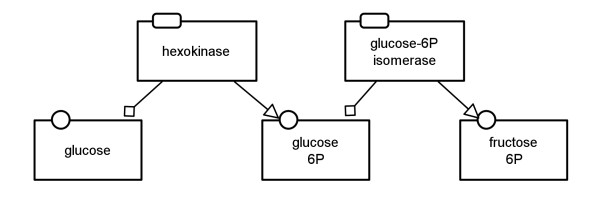
**Example of a translation to AF with the enzyme activities template applied (see also Figure **13** middle).** This map corresponds to the PD map in Figure 11.

**Figure 13:**
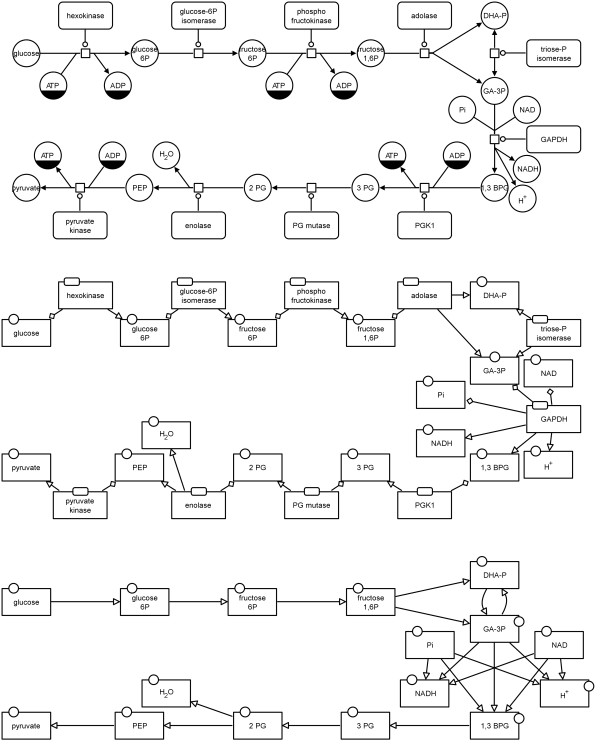
**Translation of a PD map to an AF map using the example of glycolysis from **[11]** (see also Additional file **1**).** Top: initial PD map, middle: translated AF map applying the "enzyme activities" template, bottom: translated AF map applying the "metabolite activities" template.

Figure 12 shows the AF translation of the map in Figure 11. The map in the middle of Figure 13 demonstrates how the glycolysis would look when the *enzyme activities* template is applied for the translation.

#### Metabolite activities template

**Figure 14:**
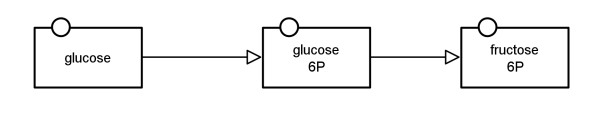
**Example of a translation to AF with the metabolite activities template applied (see also Figure **13** bottom).** This map corresponds to the PD map in Figure 11.

The *metabolite activities* template focuses on the *consumption* and *production* arcs in the network. It shows how the metabolites are converted into each other without taking into account potential modulating arcs in the system. This is the template of choice for translation if the purpose is to show only the material flow in the network. The map at the bottom of Figure 13 shows how the glycolysis would look when the *metabolite activities* template is applied for the translation. Figure 14 shows the top left part of this map which corresponds to the map in Figure 11.

#### Combined activities template

**Figure 15:**
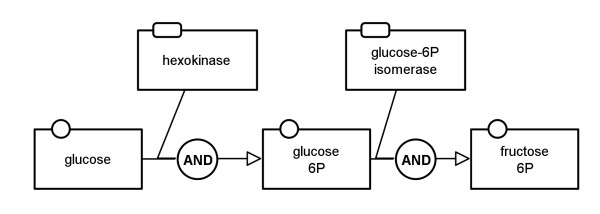
**Example of a translation to AF with the combined activities template applied (see also Figure **16** middle).** This map corresponds to the PD map in Figure 11.

**Figure 16:**
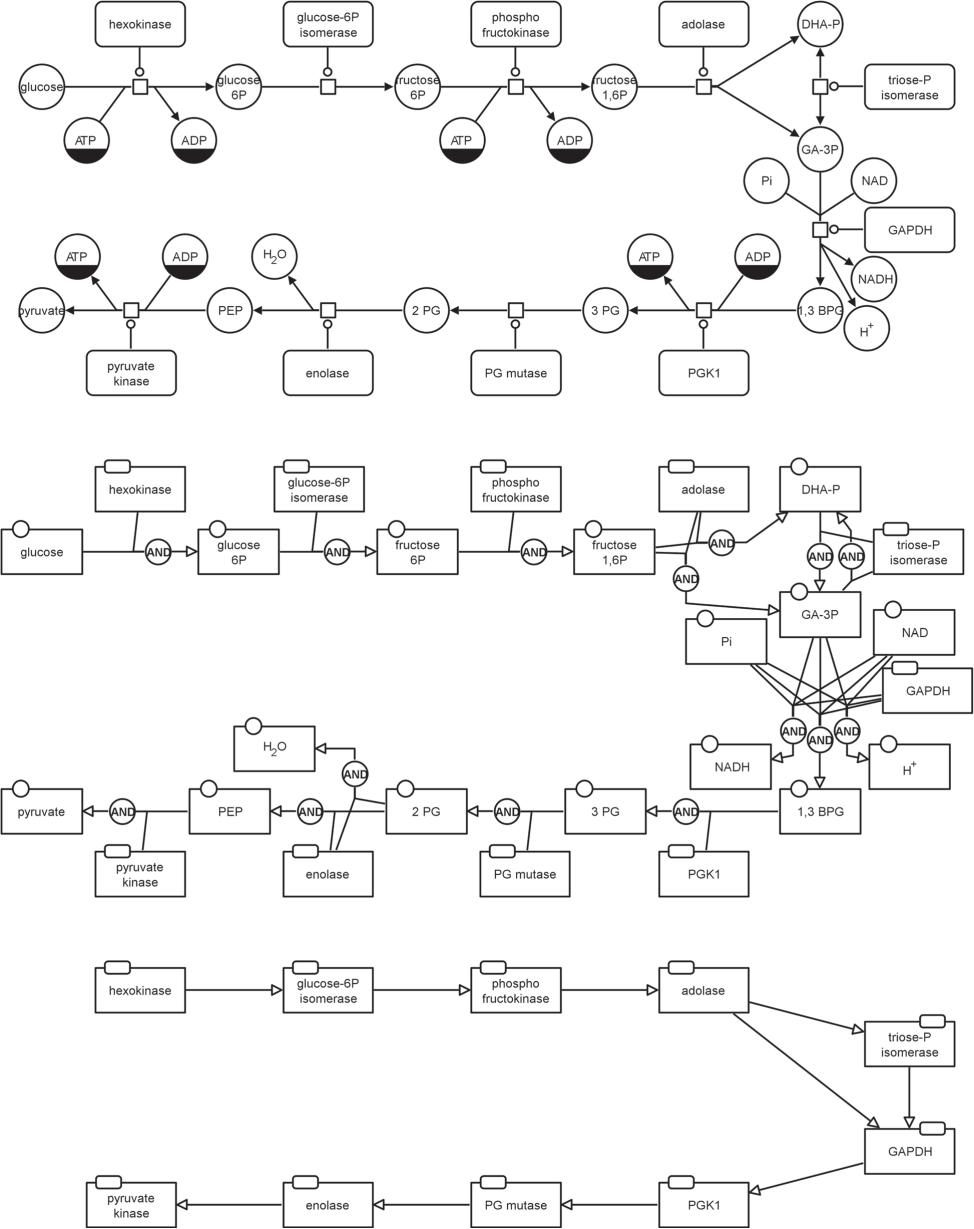
**Translation of a PD map to an AF map using the example of glycolysis from **[11]** (see also Additional file **1**).** Top: initial PD map, middle: translated AF map applying the "combined activities" template, bottom: translated AF map applying the "simple enzyme activities" template.

The *combined activities* template is a combination of the two templates described above. For this template the translation rules lead to an AF map which is very similar to the PD map since *process nodes* are basically replaced by the *logical operator and*. The only disadvantage is that *logical operators* are only allowed to have one outgoing arc. If a *process node* has more than one *production* arc, for each a *logical operator and* is added to the AF map. If a *process node* has more than one incoming modulating arc, all modulating arcs of the same type are connected to a new *logical operator or* which is then connected to a *logical operator and*. Figure 15 shows the AF translation of the map in Figure 11. The map in the middle of Figure 16 demonstrates how the glycolysis would look when the *combined activities* template is applied for the translation.

#### Simple enzyme activities template

**Figure 17:**
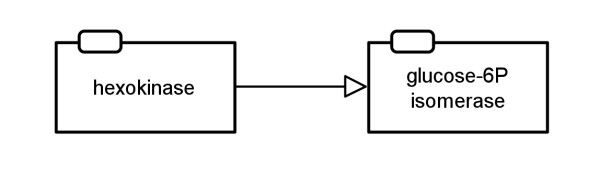
**Example of a translation to AF with the simple enzyme activities template applied (see also Figure **16** bottom).** This map corresponds to the PD map in Figure 11.

The *simple enzyme activities* template is the complementary template to the *metabolite activities* one. It shows how enzyme activities affect other enzymes without going into detail about the underlying processes. Figure 17 shows the AF translation of the map in Figure 11. The map at the bottom of Figure 16 shows how the glycolysis would look when the *simple enzyme activities* template is applied for the translation.

## Results and discussion

### Implementation

**Figure 18:**
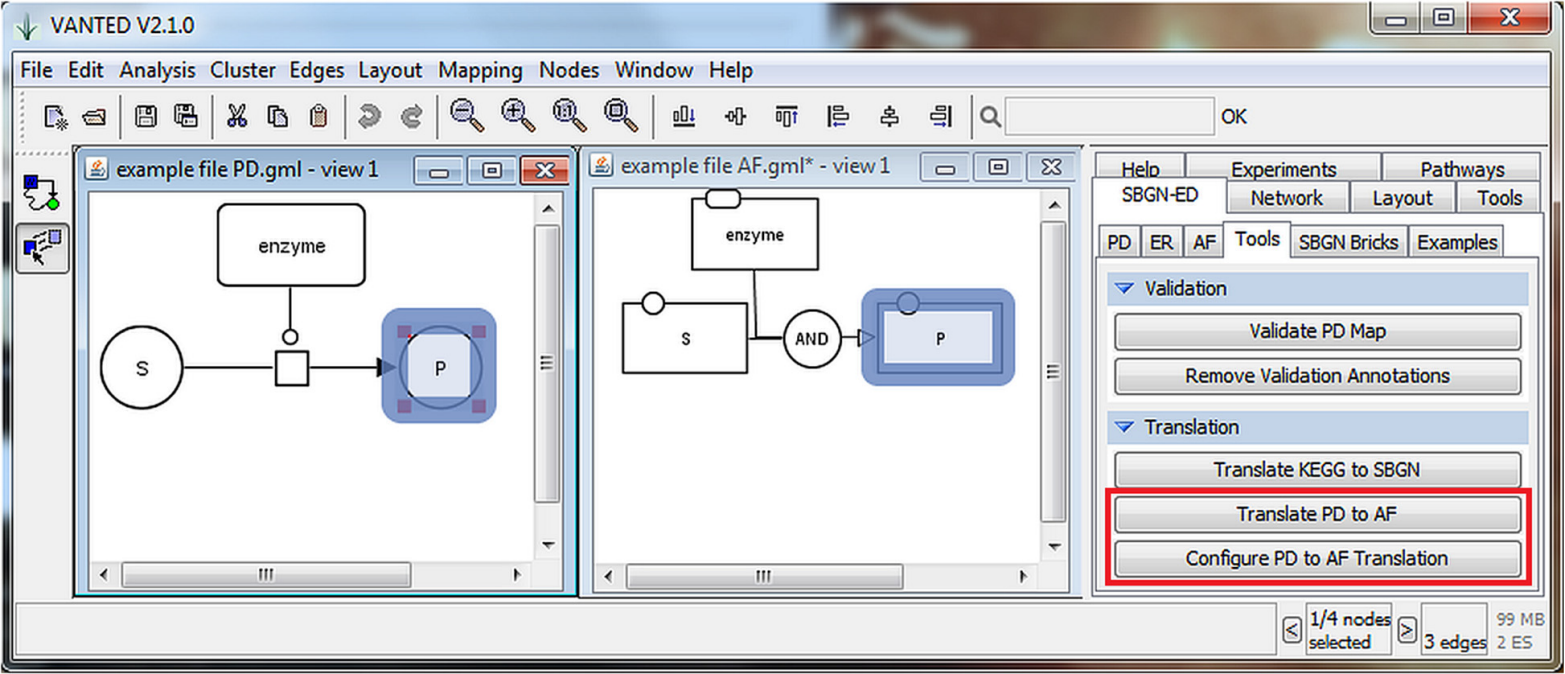
Screenshot of VANTED showing the SBGN-ED Tools tab (right) as well as a sample Process Description map (left) and the translated Activity Flow map (middle).

The translation of SBGN PD maps into SBGN AF maps is implemented as a tool in SBGN-ED ( http://www.sbgn-ed.org) (see Figure 18 for a screenshot of the system).

#### Configuration

A number of translation rules were defined earlier describing different alternatives for the translation of PD maps to AF maps. A configuration dialogue provides the user with the possibility to set up particular translation rules to create the desired AF map from a PD map.

**Figure 19:**
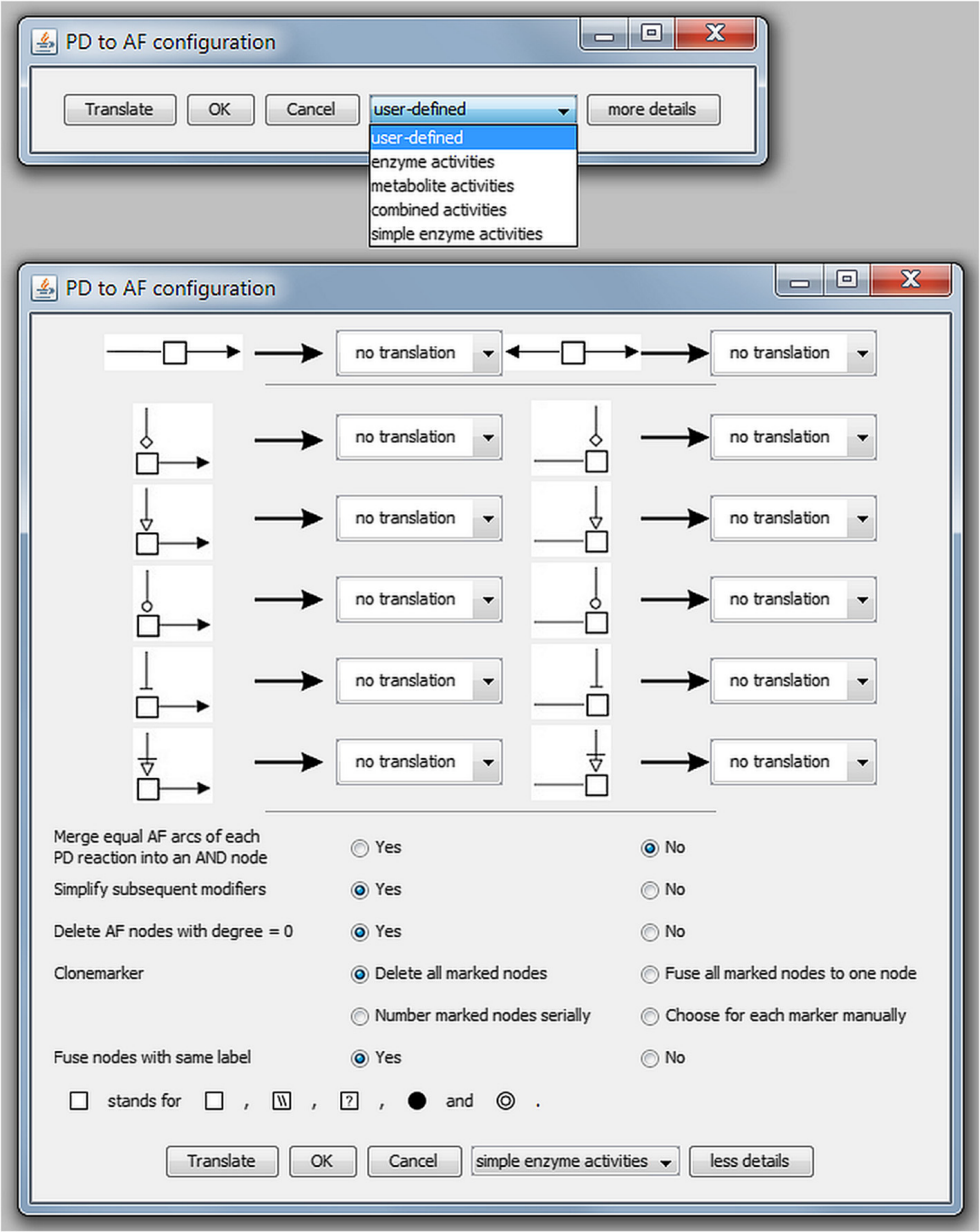
**The configuration dialog for the PD to AF translation in SBGN-ED.** The image at the top shows the default configuration dialog, the image at the bottom shows the detailed configuration dialog.

The default configuration dialog is shown at the top of Figure 19. Here the user can choose one of the translation templates (see also Translation templates). With a click on "more details" the user can expand the configuration dialog to set up a translation in more detail as shown at the bottom of Figure 19. The detailed configuration dialog shows all the defined translation rules which can now be changed/combined manually to achieve the desired result.

### Graphical representation

If the user would translate, edit and layout the AF map manually, it would be a very time consuming and intensive process. Automatic translation and layout can speed up this process. However, a problem with automatic layout is that changes of the map during the translation can disturb the layout in some manner. For example, an added arc may now cross existing arcs or two nodes may overlap. Straightforward application of an existing layout algorithm to the map for the purpose of eliminating these overlaps may totally change the layout, thereby destroying the user’s "mental map" [13]. The mental map can be imagined as the imprint of the map in the mind of the user. If the map is changed too much, it no longer corresponds with this mental imprint and may cause confusion. Therefore we do not apply a layout algorithm to the map but try to preserve the mental map of the PD map within the AF map.

In principle the described translation algorithm adopts all nodes in the AF map in size, position and colour as they appear in the PD map to maintain the mental map during translation. Nevertheless some exceptions exist for the positioning of nodes in the AF map. When nodes are combined to one node the average position of these nodes is calculated as the position for the new node. Another exception is the necessary duplication of *logical operators* with more than one outgoing arc. The duplicated nodes are arranged spirally around the position of the original node.

### Interaction between a PD map and the corresponding AF map

After the translation has been finished both the original PD map and the new AF map are shown in a linked view in SBGN-ED. Thus a user can select on one or more elements in the one map and obtain the corresponding elements highlighted in the other map, a method called linking & brushing. Since the translation of a PD map into an AF map is always associated with a loss of information the linked view together with the linking & brushing provides a possibility to the user to evaluate the translation result and to adapt the translation rules if necessary.

**Figure 20:**
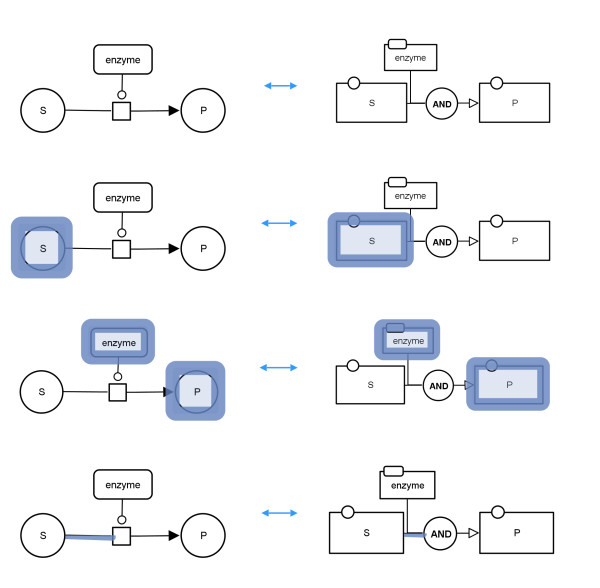
**Illustration of the interaction between a PD map (left) and the corresponding AF map (right).** From top to bottom: no selection; selected node "S" in the PD map and corresponding highlighting in the AF map; selected nodes "enzyme" and "P" in the AF map and corresponding highlighting in the PD map; selected arc in the PD map and corresponding highlighting in the AF map.

Figure 20 shows interactive highlighting between a PD map and its corresponding AF map. In the first row none of the graph elements is selected. After selection of node "S" in the PD map this node and the corresponding node "S" in the AF map are highlighted. In the third row the possibility to select more than one graph element is shown and in the fourth row the possibility to also select arcs is demonstrated.

It is possible that a PD map has links to multiple AF maps at the same time. If this PD map is translated more than once into an AF map without closing any of the AF maps, all links are preserved, and therefore linking & brushing between multiple views is possible.

### Examples

We present two examples: the translation of a metabolic network (glycolysis), and the translation of a signalling pathway (MAPK cascade).

The metabolic network in PD is shown in Figures 13 and 16 (top) and is also provided as additional file (see Additional file 1). The PD map has been translated to an AF map applying the "enzyme activities" template (see Figure 13 middle), applying the "metabolite activities" template (see Figure 13 bottom), applying the "combined activities"template (see Figure 16 middle), and applying the "simple enzyme activities" template (see Figure 16 bottom).

**Figure 21:**
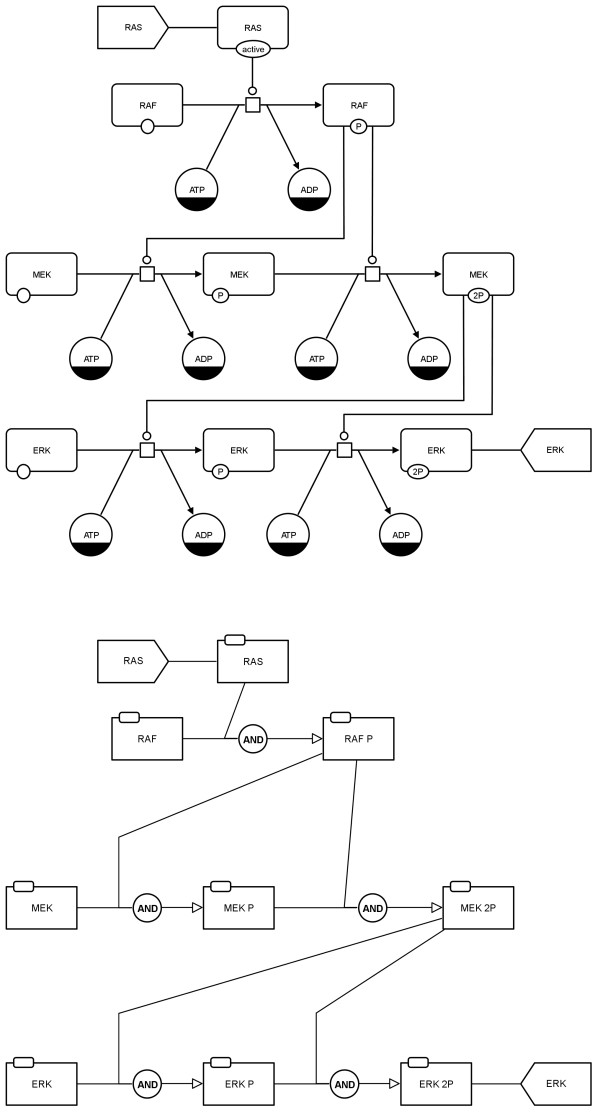
**Translation of a PD map to an AF map using the example of MAPK cascade from **[11]** (see also Additional file **2**).** Top: initial PD map, bottom: translated AF map applying the "combined activities" template.

**Figure 22:**
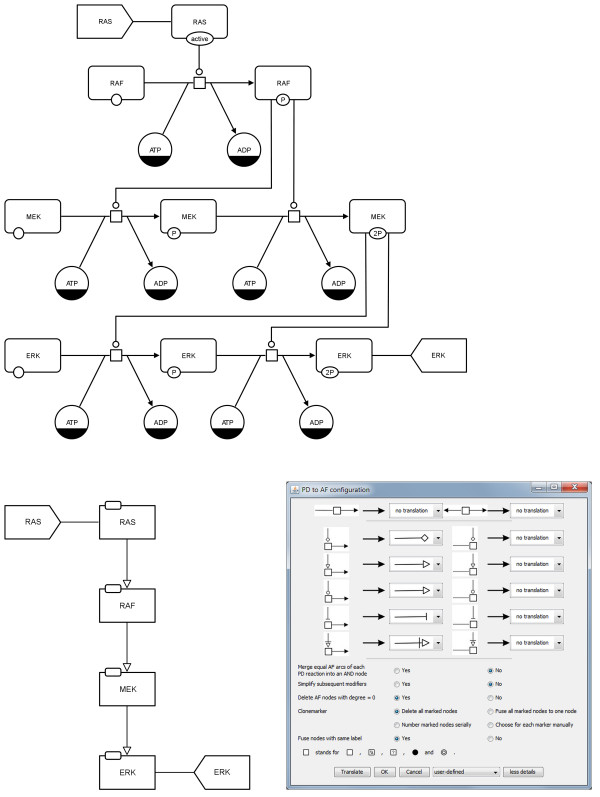
**Translation of a PD map to an AF map using the example of MAPK cascade from **[11]** (see also Additional file **2**).** Top: initial PD map, bottom left: translated AF map, bottom right: screenshot of the configuration dialog for this translation. The translation rules have been set manually.

The signalling network in PD is shown in Figures 21 and 22 (top) and is also provided as additional file (see Additional file 2). The PD map has been translated to an AF map applying the "combined activities" template (see Figure 21 bottom), and setting the translation rules manually (see Figure 22 bottom).

Another example for a large regulatory network is provided as additional file (see Additional files 3 and 4) [14].

## Conclusion

In this paper we presented methods for translating SBGN PD maps into AF maps and for interacting between these maps. As shown the complex PD maps become much smaller and more manageable when translated into AF. To navigate easily between these maps the brushing method is the tool of choice. With this highlighting it is very easy to see which part of one map corresponds to which part of the other map. These methods are integrated in SBGN-ED.

The translation of PD into AF is an important but also only an initial step in translating between different SBGN languages. We hope that our work inspires research on translation methods between other SBGN languages.

## Endnote

^a^ For a list of SBGN supporting software see http://www.sbgn.org/SBGN_Software.

## Competing interests

The authors declare that they have no competing interests.

## Authors’ contributions

TV implemented the PD to AF translation in SBGN-ED. TC advised about the implementation. FS supervised the project. All authors contributed to the intellectual design of the described techniques and contributed to writing the paper. All authors read and approved the final manuscript.

## Supplementary Material

Additional file 1 **Example file glycolysis.** This file provides the SBGN PD map for glycolysis used for the PD to AF translations shown in Figures 13 and 16. Click here for file

Additional file 2 **Example file MAPK cascade.** This file provides the SBGN PD map for MAPK cascade used for the PD to AF translations shown in Figures 21 and 22. Click here for file

Additional file 3 **Translation of the "LEC1/AFL-B3 factors and maturation gene control" map from the RIMAS database **[14]** (see also Additional file **4**).** Top: initial PD map, bottom: translated AF map. The translation rules were based on the rules provided by the "enzyme activities" template. Additionally the option "Fuse all marked nodes to one node" was activated. Click here for file

Additional file 4 **Example file "LEC1/AFL-B3 factors and maturation gene control".** This file provides the SBGN PD map for "LEC1/AFL-B3 factors and maturation gene control" used for the PD to AF translation shown in Additional file 3. Click here for file
