# Supplementary figures and images for: Translation of SBGN maps: Process Description to Activity Flow
Source: BMC Syst Biol. 2013 Oct 31;7:115. doi: 10.1186/1752-0509-7-115 (PMC4228393; doi:10.1186/1752-0509-7-115)

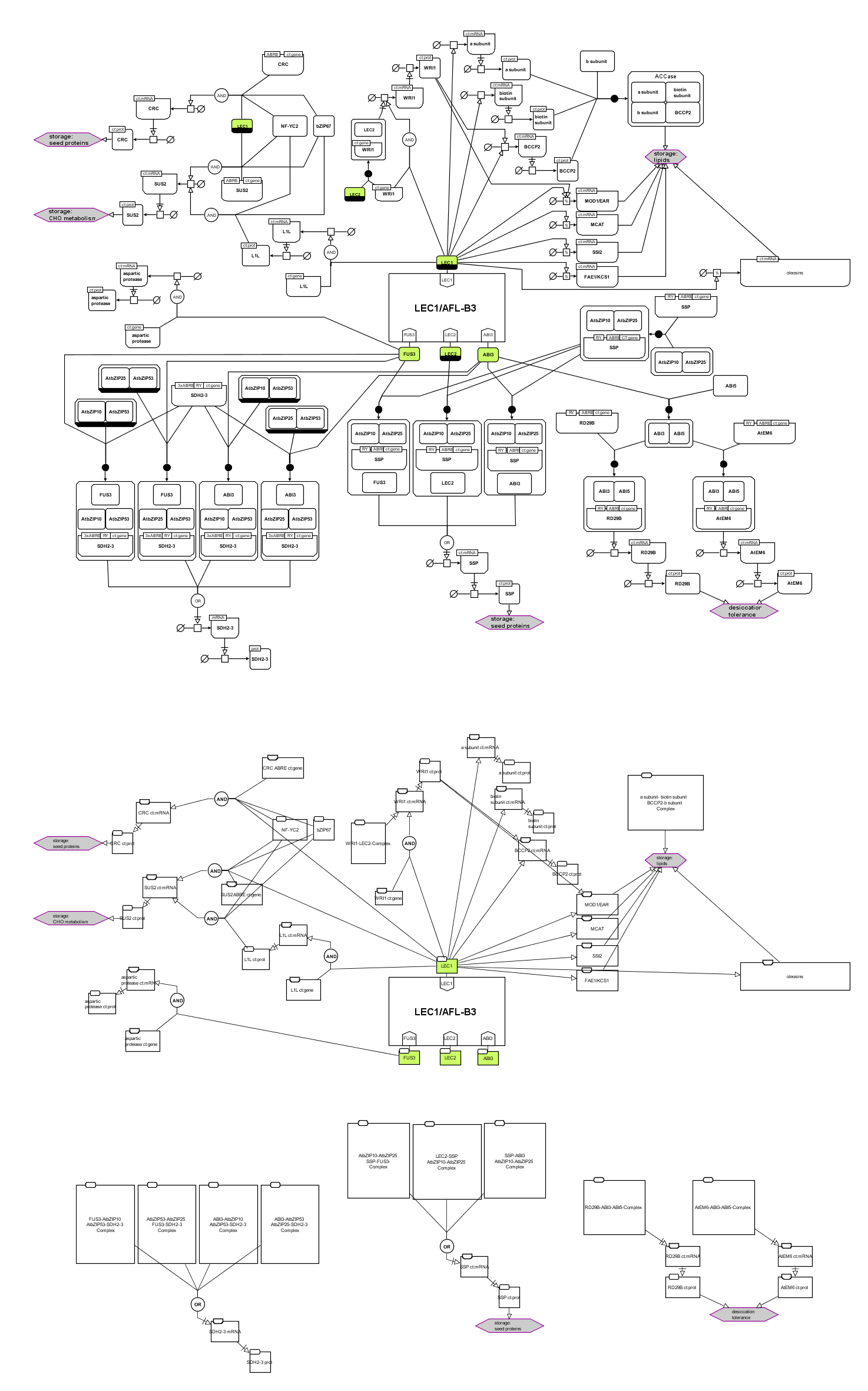

Supplement: Additional file 3 — Translation of the "LEC1/AFL-B3 factors and maturation gene control" map from the RIMAS database [14] (see also Additional file 4). Top: initial PD map, bottom: translated AF map. The translation rules were based on the rules provided by the "enzyme activities" template. Additionally the option "Fuse all marked nodes to one node" was activated. [file 1752-0509-7-115-S3.png]
